# Maternal and child health in Yushu, Qinghai Province, China

**DOI:** 10.1186/1475-9276-10-42

**Published:** 2011-10-04

**Authors:** Mary Wellhoner, Anne CC Lee, Karen Deutsch, Mariette Wiebenga, Maria Freytsis, Sonam Drogha, Phuntsok Dongdrup, Karma Lhamo, Ojen Tsering, Tseyongjee, Dawa Khandro, Luke C Mullany, Lee Weingrad

**Affiliations:** 1 Department of International Health, Johns Hopkins Bloomberg School of Public Health, 615 North Wolfe Street, Baltimore, MD 21205 USA; 2 Department of Newborn Medicine, Brigham and Women's Hospital, 75 Francis Street, Boston, MA 02115 USA; 3 Family Health Care Nursing, University of California, San Francisco, 2 Koret Way, San Francisco, CA 94143 USA; 4 Global AIDS Interfaith Alliance, Blantyre, Malawi; 5 Public Health Consultants, Zeist, Netherlands; 6 Department of Obstetrics and Gynecology, Woodhull Medical Center, 760 Broadway Brooklyn, NY 11206 USA; 7 Surmang Foundation Dharma Saghara Clinic, Yushu, Qinghai, China; 8 Department of Gesar, Unit of The Masses Arts of Yushu Tibetan Autonomous Prefecture of Qinghai, China; 9 Tibet Gzi-road Culture Communication Ltd,.Co., Xining, Qinghai, China; 10 Dronme Association for Education and Eradication of Poverty, Xining, Qinghai, China; 11 Department of Global Community Health and Behavioral Science, Tulane University School of Public Health and Tropical Medicine, 1440 Canal Street, New Orleans, LA 70112 USA; 12 Completion Primary School, Chenduo, Yushu, Qinghai, China; 13 Surmang Foundation, 13536 Gold Hill Road, Boulder, CO 80302 USA

**Keywords:** Tibetan, Qinghai, Yushu, China, facility delivery, institutional delivery, maternal morbidity, maternal mortality, maternal health, child health, newborn health, Yushu earthquake

## Abstract

**Introduction:**

Surmang, Qinghai Province is a rural nomadic Tibetan region in western China recently devastated by the 2010 Yushu earthquake; little information is available on access and coverage of maternal and child health services.

**Methods:**

A cross-sectional household survey was conducted in August 2004. 402 women of reproductive age (15-50) were interviewed regarding their pregnancy history, access to and utilization of health care, and infant and child health care practices.

**Results:**

Women's access to education was low at 15% for any formal schooling; adult female literacy was <20%. One third of women received any antenatal care during their last pregnancy. Institutional delivery and skilled birth attendance were <1%, and there were no reported cesarean deliveries. Birth was commonly attended by a female relative, and 8% of women delivered alone. Use of unsterilized instrument to cut the umbilical cord was nearly universal (94%), while coverage for tetanus toxoid immunization was only 14%. Traditional Tibetan healers were frequently sought for problems during pregnancy (70%), the postpartum period (87%), and for childhood illnesses (74%). Western medicine (61%) was preferred over Tibetan medicine (9%) for preventive antenatal care. The average time to reach a health facility was 4.3 hours. Postpartum infectious morbidity appeared to be high, but only 3% of women with postpartum problems received western medical care. 64% of recently pregnant women reported that they were very worried about dying in childbirth. The community reported 3 maternal deaths and 103 live births in the 19 months prior to the survey.

**Conclusions:**

While China is on track to achieve national Millennium Development Goal targets for maternal and child health, women and children in Surmang suffer from substantial health inequities in access to antenatal, skilled birth and postpartum care. Institutional delivery, skilled attendance and cesarean delivery are virtually inaccessible, and consequently maternal and infant morbidity and mortality are likely high. Urgent action is needed to improve access to maternal, neonatal and child health care in these marginalized populations. The reconstruction after the recent earthquake provides a unique opportunity to link this population with the health system.

## Introduction

China is on track for meeting Millennium Development Goals 4 and 5 [1-3], predominantly due to rapid development in the country's urban and densely populated regions. Success in these areas, however, may mask substantial health inequities since data are lacking from remote low-resource regions of the western provinces primarily inhabited by ethnic minorities. In general, due to geographical challenges, lack of infrastructure and low population density, the health status of China's rural minorities is poorly documented. For instance, the lack of information on the ethnic Tibetan population separate from the rest of China has historically represented an ongoing barrier to improving the health of the Tibetan people [4].

Qinghai Province in western China comprises most of the Tibetan High Plateau and is one of the poorest regions in China. In 2003 Qinghai annual per capita GDP was $1814 for urban areas and $483 for rural areas [5]. Almost half of Qinghai's population is comprised of ethnic minorities. The nomadic lifestyle and geographic isolation of these 1.5 million ethnically Tibetan people make health and demographic information difficult to obtain and health care difficult to deliver. Furthermore, their economic, political, social and educational disadvantage has translated into poor access to health care and poor health [4,6] compared to China overall.

**Figure 1:**
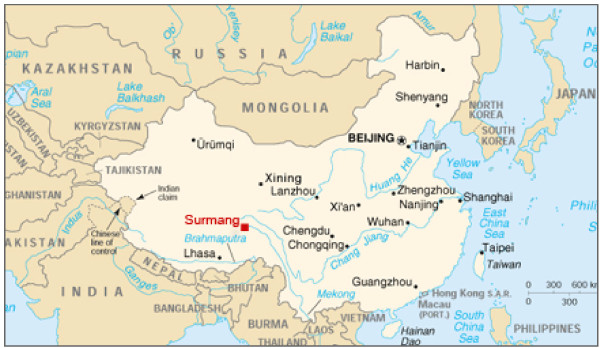
**Surmang, Yushu Prefecture, Qinghai Province**.

Xiao Surmang (Figure 1) is a sparsely populated ethnically Tibetan area in the south of the province (population ~ 7000). The township is located 95 kilometers from Yushu (population 100,000), the capital of Yushu Prefecture. On April 14, 2010 approximately 90% of the buildings and all of the government health facilities in Yushu were destroyed by a 7.1 magnitude earthquake leaving more than 3500 dead. Many of the surrounding township clinics were also damaged in the earthquake, further reducing health service delivery. As of a year after the earthquake, health services in Yushu have been reestablished in temporary facilities as hospitals are being rebuilt. The Surmang Clinic, run by the Surmang Foundation, a non-governmental organization in the area, is a primary source of western medical care for the area's rural Tibetan population. The Surmang Foundation was founded in 1992 by an American who currently resides in Beijing and is funded by a variety of donors, including private individual donors in the US and Canada, smaller online contributions, multinational corporate donors inside China, and Chinese individual and corporate philanthropy.

The original purpose of this 2004 survey was to assess maternal and child health knowledge, attitudes and practices in the remote ethnically Tibetan minority population surrounding the Surmang Clinic near the town of Yushu, in order to inform provision of local clinical services. The purpose of this paper is to present the results of this survey of 400 local women and to highlight the health inequity suffered by the population compared with other segments of the Chinese population and with China overall. Furthermore, since the town of Yushu and the local health infrastructure were destroyed by the earthquake, this data becomes even more relevant, as the reconstruction efforts provide an opportunity to reduce these inequities. In addition to aiding reconstruction of the health system after the earthquake, such documentation of the health services gap among one of China's most disadvantaged and vulnerable populations -- western province, rural, minority women and children -- is urgently needed and will assist the Chinese government to direct appropriately targeted resources to achieve its health reform goal of a "Healthy China by 2020."[7]

## Methods

In August 2004 the Surmang Foundation collaborated with the Yushu County Department of Maternal and Child Health to conduct household surveys of reproductive age women (15-50 years) among the sparse nomadic population residing in Surmang Clinic's surrounding catchment area. The population and distribution of inhabitants in the region were initially identified and enumerated in two mapping exercises of surrounding villages and nomadic areas in consultation with village and community leaders (June-July 2004).

### Instrument Development

The survey tool was adapted initially from the Nepal Reproductive Health Survey [8] and another similar regional survey [9]. The instrument was refined for contextual appropriateness and additional content added based on several informal focus group discussions with local women (n = 5-10/group) regarding pregnancy, childbirth and infant health. Three villages were chosen for focus groups, all in close proximity to the Surmang Clinic, and all with less than 12 women currently residing in the village. Women present in the villages on the day of the focus groups were asked to participate, and a total of 22 women agreed. All 22 women interviewed were farmers. Participation was on a voluntary basis, and only one woman of those approached declined to participate. Those villages used for focus groups were excluded from the subsequent survey sample.

The focus group script was created in English and translated to Tibetan. One of the 22 women was interviewed separately as a key informant in creating the focus group script. Two female project staff conducted the groups; one fluent in Chinese and local Tibetan dialect and another fluent in Chinese, local Tibetan and English. While the first woman led the focus groups in Kham Tibetan, the second took notes in Chinese, which were then translated by her into English for analysis. The focus group script included open-ended questions regarding normal self-care during pregnancy and birth as well as recognition and management of complications. There were also questions regarding common causes of childhood illness, their management and overall care seeking behavior for pregnancy, birth, babies and children. Prompts for the focus group leader where included to help elicit relevant information.

The survey itself was primarily a closed-ended questionnaire containing sections on socio-demographic information, marital and reproductive history, current and prior pregnancy histories, prior delivery history, postpartum history, infant care and health at birth, breastfeeding and complementary foods, child health, access to health facilities and maternal mortality. Since this was a self-reported retrospective survey of women with very little access to health care, no medical verification of their self-reported symptoms, problems or complications was available. Most of the questions regarding symptoms and problems included a short list of possible problems, and the woman was asked to respond yes or no to each item in the list.

#### A typical question regarding pregnancy problems

Which of the following problems did you have during your last pregnancy? Did you have... (*read aloud*)

1. Vaginal bleeding? Yes No DK NR  

2. Swelling in face, hands and feet (ALL 3)? Yes No DK NR  

3. Fever? Yes No DK NR  

4. Headache? Yes No DK NR  

5. Dizziness and blurry vision (BOTH)? Yes No DK NR  

6. Abdominal Pain? Yes No DK NR

#### And a typical question regarding postpartum problems

Did you have any of the following problems within one to two months (42 days) of your last child's birth... *(read all responses aloud)*

1. Excessive vaginal bleeding ? Yes No DK NR  

2. Fever? Yes No DK NR  

3. Foul smelling vaginal discharge? Yes No DK NR  

4. Severe pain in the lower abdomen? Yes No DK NR  

5. Burning when you urinate? Yes No DK NR  

6. Painful red infected area in breast? Yes No DK NR  

7. Feelings of depression? Yes No DK NR

The survey included the taking of a complete pregnancy history from each woman, including year of delivery for each pregnancy, her age at delivery, pregnancy outcome (such as live birth, still birth, spontaneous or induced abortion), gestational age, gender, location of delivery and attendant, current age and status of the child and cause of death. Questions were originally written in English, translated to Tibetan, and back translated to English for verification purposes. The survey tool was tested with 10 Kham women in surrounding communities. During the pilot phase, the women were asked about their understanding of the specific questions, and wording of unclear terminology or phrasing was improved. Though efforts were made to use simple local terminology, pretest on community women and check content with back translation, the very low level of education seen amongst the women may have affected their ability to understand and answer questions. Answers to questions regarding subjective complaints during pregnancy and childbirth were also subject to recall bias. The survey required approximately 30 minutes to complete.

### Training

Interviews were conducted by eight Kham women who were literate and fluent in the local Kham dialect. Two Tibetan clinic physicians trained the interviewers over a 2-day period, reviewing individual question content and responses, survey skip patterns, data entry, and communication techniques. Each interviewer conducted 4-5 test interviews among fellow trainees before beginning fieldwork.

### Sampling

The target sample included all women of reproductive age within accessible regions of the clinic catchment area. Accessible areas were selected based on travel time within 6 hours from the Surmang Clinic by vehicle, horseback or on foot. Given the nomadic lifestyle and the rigorous Himalayan terrain, challenges arose in mapping and enumerating all potential inhabitants in the region; however, extensive efforts were made to include women in difficult to access areas, such as inhabitants of cave-dwellings and pastoralists in high pasture lands. The number of women of reproductive age was enumerated based on the surveyors' interviews with village-family leaders and estimated to be around 600.

### Data Collection and Analysis

Oral consent was obtained prior to conducting the interview. Data was entered into an SPSS database, later converted to Stata, then reviewed and cleaned of outlying values. Unique identifiers were eliminated. Continuous data was summarized by mean and standard deviation if normally distributed, otherwise by median and range. Categorical data was summarized by simple frequency tabulation.

### Ethical Approval

Approval to conduct the survey was provided by the Yushu Department of Public Health and Maternal and Child Health and the Beijing United Family Hospital Ethics Review Board; the Institutional Review Board of the Johns Hopkins Bloomberg School of Public Health provided approval for data analysis.

## Results

Based on village visits and mapping exercises, 389 households and approximately 606 women were identified during the two months prior to the survey. In August 2004, 470 women were approached and 409 women (87%) agreed to participate and completed the survey. Common reasons for declining to participate in the study included that a different dialect of Kham Tibetan was spoken or that the woman was too busy with chores or physical labor. Given the nature of nomadic populations, not all of the originally enumerated women were located at the time of the survey, as some would have moved to higher pastures or different dwellings between the time of mapping efforts and the time of the survey. Most of the interviews (78%) took place in village housing, and the remainder in nomadic tents. Seven interviews were excluded because the respondent was not within the target age range (15-50 years), leaving 402 interviews in the analytic data set.

The mean age of respondents was 30.1 years (SD = 8.8) and all were Tibetan, from the Kham region, and Buddhist (Table 1). Seventy-seven percent were either currently or ever married; among these the mean age at marriage was 22.5 (range 15-35) years, and 19% were married before age 20 years. Educational attainment and literacy were low, with 15% (n = 59/399) reporting any formal education, only 2% reading easily and 20% reading with difficulty. Only one respondent reported using a latrine/toilet; the majority (98%) reported defecating in an open field or in no designated area, and only rarely (n = 5, 1%) was hand washing considered important after urinating/defecating. One third of respondents reported using both soap and water when they do wash their hands. The average time to reach a health facility was 4.3 hours (range 0.5 minutes-9 days). Common modes of transport included walking (41%), motorcycle (25%), horseback (25%), and car/truck (3%). For the 5% of women (n = 19) who reported their travel time to medical care in days, the majority would be on horseback (53%), followed by motorcycle (18%), walking (17%) and truck (12%).

**Table 1:** Population Characteristics

Characteristic	N	%
**Age in Years (mean/SD)**	30.1	8.8

**Ethnicity**		

Tibetan from Kham region	392^1^	100

**Religious affiliation**		

Buddhist	401^2^	100

**Age of marriage (mean 22.5, sd 3.7)**		

<18	21	9

18-24	138	60

25-29	60	26

> = 30	10	4

**Marital Status**		

Married	232	60

Single, never married	91	24

Single with children	51	13

Divorced, widowed or separated	10	4

**School Completion Rates**		

Primary	53	13

Secondary	6	1

**Maternal Literacy**		

Unable to read at all	252	63

Reading with difficulty	75	19

Reading easily	8	2

**Women's Occupation**		

Nomad	213	53

Farmer	66	17

Housewife	63	16

Nomad and farmer	49	12

Other	10	2

**Husband's Occupation**		

Nomad	90	39

Farmer	64	28

Driver	34	15

Logger	8	3

Business	7	3

Other	25	11

**Primary Sources of Household Income**		

Caterpillar fungus^3^	234	59

Sale of animals	29	7

Animal products	19	5

Husband's income	20	5

Temporary contracts	6	2

Other	87	23

^1^10 missing; ^2^1 missing; ^3^Caterpillar fungus is a highly valued traditional Tibetan medicine harvested from the Tibetan Plateau and is an important source of income for the population.

Two hundred and seventy (67%) women reported ever being pregnant; this included 26 (9%) who were currently pregnant and 233 (60%) who reported a pregnancy in the last five years and whom were asked a series of questions specifically about their last pregnancy and delivery.

### Antenatal, Intrapartum, and Postpartum Care

Over half of women (52%, 122/236) who had been pregnant in the last five years reported seeking preventive care during their last pregnancy. Their preferred providers included western doctors (62%), lamas or monks (41%), and traditional Tibetan doctors (9%) (multiple providers reported per woman). Reasons for not seeking care included distance (32%), lack of perceived necessity (32%), financial constraints (11%) or unavailability (9%); yet most women (93%) indicated they would attend antenatal care if it were available near their village. Only 14% of respondents received tetanus toxoid during their last pregnancy. Among these, a single injection (73%) was more commonly reported than multiple injections (27%).

The majority of the women (81%) did not reduce their physical workload during pregnancy. The Tibetan women in this area do heavy physical work for many hours a day. For nomadic women this includes milking yaks several times daily starting at 4 or 5 am, churning butter, making yogurt, spinning wool to make clothing and materials, tending the children and yaks, collecting and preparing yak dung for fuel and cooking meals. For women in the townships, the lifestyle is agrarian; working in barley or turnip fields daily and walking up into the highlands to harvest herbs, in addition to the duties of cooking, cleaning and tending the children. Water must be carried from the river for drinking and cooking for both nomadic and agrarian Tibetans, and all the washing and cleaning is done in the river by women.

Substantial proportions of women were very (64%), somewhat (9%), or a little (9%) worried about dying during childbirth. Facility delivery was extremely rare (0.2%); women more commonly delivered at home, usually in a house (85%), a tent (10%), or an animal shed (4%). Almost all (99%) births occurred without a skilled birth attendant. Common attendants included mother or mother-in-law (65%), another female relative (12%), husband (10%), Tibetan doctor (6%), father (5%), or traditional birth attendant (2%). Nineteen women (8%) delivered alone. One woman was attended by a western doctor, and none were delivered by cesarean section. In the first week after delivery, 95% of mothers did not bathe and only about 3% bathed with soap and water. The mean postpartum recovery period before starting work again was 10.6 days, with a range of 0-60 days.

### Self-Reported Symptoms and Care-seeking

Self-reported problems during the antenatal, intrapartum, and post-partum periods were common (Table 2). Almost half of respondents self-reported one or more antenatal problem or symptom, including dizziness and blurry vision (48%), headache (46%), abdominal pain (41%) or fever (40%). Care for such problems was most likely sought from a Tibetan medicine doctor (70%), family, neighbors or friends (14%), western medicine doctor (9%), or lama or monk (7%). Self-reported complications during labor and delivery were also common, with 37% of women reporting prolonged labor, 17% excessive bleeding, 9% retained placenta ("placenta stuck inside"), 7% obstructed labor ("baby would not come out after pushing"), and 27% seizures. Women also commonly reported postpartum symptoms such as severe lower abdominal pain (56%), fever (42%), mastitis ("painful red infected area in breast") (41%), excessive bleeding (29%), foul-smelling vaginal discharge (27%) and dysuria ("burning when you urinate") (24%). Care seeking for postpartum complaints was uncommon; overall only 38% of affected women sought help, usually from a traditional Tibetan doctor (87%), and less commonly from a western medicine doctor (7%).

**Table 2:** Prevalence of Maternal Symptoms during antenatal, intrapartum and postpartum periods

	N	Percent (%)
**Antenatal**		

Dizziness and blurry vision	111	48

Headache	107	46

Abdominal pain	96	41

Fever	93	40

Swelling in face, hands and feet	64	27

Vaginal bleeding	13	6

**Intrapartum**		

Prolonged labor	79	37

Seizures	58	27

Excessive vaginal bleeding	37	17

Retained placenta ("placenta stuck inside")	19	9

Obstructed labor ("baby would not come out after pushing")	14	7

High blood pressure	6	3

Vaginal tear	5	2

**Postpartum**		

Severe lower abdominal pain	132	56

Fever	99	42

Painful/red/infected breast	95	41

Postpartum depression ("feelings of depression")	71	31

Excessive vaginal bleeding	66	29

Foul-smelling vaginal discharge	61	27

Dysuria ("burning when you urinate")	54	24

When the women were asked if they knew of anyone in their village who had died in childbirth or up to six weeks postpartum in the past year, 25 responded that they did know of a maternal death. Further questioning regarding name, date, location and cause of death revealed that they were all reporting the same three maternal deaths, but one of the deaths was 15 months prior to the survey. All three mothers died at home. According to the respondents, one of the mothers died from bleeding and retained placenta, and cause of death of the other two is unclear.

Live births in the interview population were estimated from the complete pregnancy history elicited from each woman, a total of 980 pregnancies among the 402 women. The accuracy of this data is somewhat limited by inconsistencies and in some cases by missing data. Dates of birth were often recorded in animal year, and time of the new year varies amongst Tibetan, Chinese and western calendars. While current age of the child should confirm date of birth, it is common in Asian cultures to consider a baby one year old at birth. Recording of both the mother's current age and age at delivery was somewhat helpful in confirming recent live births, however. Since only year of birth was recorded, live births were counted from the beginning of 2003 up to the time of the survey in August 2004, a 19-month time period roughly corresponding to the time period for which maternal deaths were reported. Liberal inclusion criteria revealed 103 live births among the 402 respondents in this 19-month time period. Given the imprecision of the dates, inability to corroborate the deaths, small sample size and uncertainty of truly capturing all live births in the study population, a maternal mortality ratio was not calculated. While all recent maternal deaths in the population were probably captured by the survey, live births among those who declined interview and those not located may have been missed.

### Neonatal and Infant Care Practices

Information on newborn and infant care practices for the most recent live birth was available among 233 (60%) women. The umbilical cord was nearly universally (94%) cut with an unsterilized instrument, usually a knife or scissors, and then tied with string. According to the mothers' estimate and recollection, approximately 9% of infants failed to initiate breathing within five minutes after birth, and 15% of newborns emerged "floppy or limp." Breathing was aided by rubbing the baby (50%), sucking the mucus from the baby's mouth and nose (7%), rescue breathing (4%), and "other" means (20%). Methods for neonatal temperature maintenance included wrapping in animal skin (58%), placing in a jacket (20%) or blanket (17%). While 85% of women reported ever breastfeeding their infant, pre-lacteal feeds were common (46%), and first foods included butter (28%) or animal milk (8%). Colostrum was commonly given (91%), and 58% of neonates initiated breastfeeding in the first two hours, another 10% on the first day, 22% between two and three days, and 10% more than three days after birth. Delayed breastfeeding was attributed primarily to lack of milk (79%) and fatigue (12%). Breastfeeding continued for an average of 24 months, and complementary feeding was delayed (77% and 12% beyond 6 and 18 months, respectively).

### Child Health

One hundred ninety-one women reported caring for children under the age of 5. In these children, the prevalence of diarrhea in the 2 weeks preceding the survey was 25% (n = 56), with a prevalence of bloody diarrhea of 4% (n = 7). Among these households, 36% reported seeking care: 73% from a traditional Tibetan doctor, 30% western medicine doctor, 8% Monk/Lama, and a few from family and friends. Cough in the prior 2 weeks was reported among 28% (n = 64) of children. Sixty percent of these mothers sought advice or treatment outside the home: 74% from a traditional Tibetan doctor, 30% from a western medicine doctor, and 6% from a family member. Average developmental milestones reported by mothers included walking without assistance at 16 months (range 9-36 months), and talking at 26 months (range 2-60 months).

## Discussion

This survey documents self-reported health determinants in the Tibetan population of Surmang --poverty, low female education and literacy, lack of sanitation, transportation and infrastructure and lack of access to basic maternal, neonatal and child health care - which are often associated with poor health. Women everywhere are entitled to skilled care at delivery as a universal human right, and no woman should die giving life. The majority of women in Surmang were very worried about dying in childbirth, and the report of 3 recent maternal deaths in this small population suggests exceedingly high maternal mortality in this region.

Facility delivery rates <1% in Surmang in the 5 years between 1999 and 2004 can be compared with facility delivery rates of 92% for China overall [10], 98.5% facility delivery for eastern provinces [1], 66.6% for western regions [1] and 34% for Qinghai Province [11]. Strong correlation between facility delivery rates and maternal mortality ratio (MMR) has been demonstrated throughout China, with highest official figures being MMR of 467 (TAR 2000) corresponding to 20% facility delivery [1]. This survey was not intended to and is not powered to adequately estimate MMR in the Surmang area. However, the facility delivery rate of <1% reliably demonstrated in the current survey would likely produce a substantially higher MMR than the highest official MMR of 467 associated with 20% facility delivery in TAR. While the results of this survey cannot accurately estimate MMR in the Surmang subpopulation, they do suggest very high maternal mortality by global standards. Skilled care and cesarean delivery (both <1%) were also virtually inaccessible, which is unacceptable by global and Chinese standards. Population cesarean section rates less than 1% are associated with maternal death, as well as excess intrapartum fetal loss and neonatal morbidity and mortality [12].

The three classic delays in obstetric care which lead to maternal mortality are discussed below as they apply to the Surmang setting. As of 2004, the three delays in Surmang were not delays but nearly complete barriers to care, even before the recent earthquake destroyed the emergency obstetric referral system.

### Barriers to Seeking Care

Female education and literacy rates well below Qinghai and national standards limit illness recognition and demand for health and maternity care. Cultural concepts of a safe delivery, such as avoiding contact with harmful spirits by staying home [13] and delivering in unclean areas [6], can only be countered by education. Improved education for girls and women would likely increase female empowerment, improve health care utilization and delay marriage and childbearing.

Despite this avoidance of facility delivery, over half of Surmang women reported seeking preventive care during their last pregnancies, about a third from a western medicine doctor. The vast majority said they would go for antenatal care if it were available in their village; cost and convenient access were limiting factors. Improving availability and affordability of these services is an appropriate next step toward improving uptake of facility delivery. In contrast to preventive antenatal care, the women overwhelmingly preferred Tibetan medicine to western medicine for problems or illness during pregnancy, possibly because of easier access at the village level. Incorporating local Tibetan medicine providers into the health system may improve utilization of services and encourage appropriate referral.

### Barriers to Reaching Health Facility

Geographic factors and distance are strong predictors of health care utilization, and lack of transport is a common preventable cause of death for both mothers and newborns [14,15]. The geographic isolation and rugged terrain, lack of reliable transportation for mothers in labor (most often by horse or motorcycle), poor road infrastructure and challenging weather of the Tibetan Highlands represent significant barriers to skilled delivery care in this population. None of the mothers in Surmang met WHO's maternal health access criterion of one hour to EmOC [16], and access has worsened since the earthquake. However, facility delivery rates were no better in another survey of Qinghai Tibetans where physical access to facility was much easier [17], potentially indicating cultural, political, social and economic factors as barriers to care in the population, as well as lack of physical access to delivery facilities.

### Barriers to Adequate Care at Facility

Since <1% of Surmang women delivered in facility, the third delay of inadequate care at facility could not be evaluated by this report. The two government hospitals providing EmOC in Yushu were completely destroyed in the earthquake and have now been reestablished in temporary facilities. Outside Yushu town, some primary level health village clinics provide skilled care, but very few women access this care. In the NGO-run Surmang Clinic some increase in facility delivery has been observed in recent years [18].

The self-reported prevalence of symptoms commonly associated with maternal morbidity is high in this population, highlighting the need for skilled delivery care. Almost half of mothers subjectively experienced prolonged and possibly obstructed labor, while 17% felt they had excessive bleeding during delivery. Medical confirmation of these complications was not possible, and some problems may be over-reported due to varying definitions, poor recall and language challenges. For instance, the self-reported prevalence of seizures of 27% was clearly excessive, most likely reflecting misinterpretation of the term. However, almost half of women had antenatal complaints of dizziness/blurry vision and headache and over a quarter complained of swelling, all of which is concerning for undiagnosed and untreated preeclampsia, warranting further study.

Postpartum complaints were also frequent -- 56% reporting severe lower abdominal pain, 42% fever, 41% possible mastitis, 29% excessive bleeding, 27% foul discharge and 24% dysuria -- but only 38% of women with postpartum complaints sought care. These symptoms could indicate high rates of infectious complications, but less than 3% of women with self-assessed postpartum problems obtained western medical treatment, again highlighting the need for skilled care in the postpartum period as well. Again, the women strongly preferred local Tibetan medicine for these problems possibly because traveling out of the home soon after birth is proscribed in Tibetan culture, further limiting access to western medical treatment.

The poor sanitation-hygiene and lack of access to clean water may be contributing to the high rates of childhood diarrhea reported in the population. Lack of access to sufficient water is probably the main reason 95% of mothers did not bathe in the first week postpartum, and this could also contribute to maternal infectious morbidity. A clean instrument was used to cut the umbilical cord in only 6% of deliveries. In this setting, clean delivery is a priority, low-cost intervention that may be achieved with clean delivery kits and behavior change messages implemented by health workers and/or family members. The Surmang tetanus vaccination rate less than 14% requires urgent action. This is a low-cost highly effective intervention for reducing both neonatal [19] and maternal tetanus and may have substantial protective benefit for this population where women often deliver on yak dung or a dirt floor and choose the animal shed or another unclean part of the dwelling for delivery [6,17].

Although breastfeeding rates and duration are high in all Tibetan population surveys, Surmang women reported delays in initiation of breastfeeding and high rates of non-exclusive breastfeeding, which have been associated with higher infant morbidity and mortality [20]. Age of introduction of complementary feeding in Surmang was significantly delayed compared with other surveys [17,21], and all available studies of the rural Tibetan population indicate complementary food is low in protein and vegetable variety compared with Chinese urban infants [21]. These nutritional deficits and late weaning could contribute to the high prevalence of childhood stunting in the Tibetan Plateau population [22], which can, in turn, lead to obstructed labor [23].

The devastation of the recent earthquake provides an opportunity for both government and local NGOs to reevaluate the existing health system and consider ways of improving delivery of maternal and child health services in this marginalized Tibetan population. In addition to strengthening health facilities, innovative strategies need to be considered to improve demand for skilled delivery care and to link the poorest mothers and newborns with the health system. In Yushu, conditional cash transfers were instituted to pay mothers 300 yuan per hospital delivery; follow up evaluation of the impact of this program is needed. Community mobilization, and female empowerment via women's participatory groups can increase skilled care at delivery by as much as 50% as reported in a recent meta-analysis [24]. Other effective strategies for improving uptake of skilled delivery care include community based loans, referral and transportation schemes or radio/cell phone communication systems, which can be used in remote low-resource settings for triage and coordination of transport [24].

In this setting, decades may be required to achieve universal facility access, and there may be a role for parallel community-level interventions alongside health facility strengthening. Community health workers (CHWs) can assist with tetanus and childhood vaccination, maternal nutrition, antenatal care, identification of danger signs during pregnancy and delivery, clean delivery, adequate care of the umbilical cord, timely initiation of breastfeeding and improved introduction of complementary feeding. CHW programs have been effective in reducing neonatal mortality from "birth asphyxia" and infections [25-27]. A package of services provided by CHWs can reduce early neonatal mortality by 36% [28]. CHWs can also be trained to successfully assess and treat infant and childhood illnesses such as pneumonia, further reducing child mortality [29]. For prevention of maternal mortality, evidence suggests that local provision of medications for postpartum hemorrhage and sepsis by CHWs can reduce maternal morbidity and mortality over and above health facility strengthening alone [30-32]. While community level interventions are controversial for maternal health, they need to be considered and evaluated as a bridging mechanism in populations such as Surmang that are not being reached by the health system.

### Limitations

The small sample size was not powered for estimating neonatal, child, or maternal mortality rates. Though care was taken to prepare and execute the survey using local language, there was misclassification of some birth outcomes (fetal deaths, stillbirths, neonatal deaths and miscarriage) that may have reflected translation issues, and thus it was difficult to interpret pregnancy outcome data. Furthermore, all data was based on self-report, which is subject to reporting and recall bias. Though care was taken to use the most appropriate local terminology for medical symptoms, understanding may have been limited by low education and varying definitions. No information was obtained on contraceptive prevalence and practices, one of the pillars of safe motherhood. Water quality and quantity was not directly assessed but likely contributes to the poor hygiene and infectious morbidity documented by the survey.

## Conclusions

The vast, isolated, mountainous regions of China's western provinces are home to millions of China's poorest citizens. Providing health care to these isolated populations while preserving their traditional culture and lifestyle is one of the greatest public health challenges in the world today. Lack of education, poor sanitation, poverty, cultural factors, geographic barriers, inability to afford medical care and lack of adequate health care facilities are all barriers the women of Surmang face in achieving safe motherhood and healthy families. The rates of facility delivery and skilled care at delivery seen here are as low as anywhere in the world, most likely resulting in extremely high maternal and neonatal mortality. Since the Surmang women were suffering from a complete lack of EmOC even before the recent earthquake destroyed the emergency obstetric referral system, reconstruction alone will not resolve this disparity.

Recent evidence suggests that an equity-based strategy aimed at improving the lives and health of the most impoverished populations is the most cost effective approach for achieving the UN Millennium Development Goals [33]. Active government participation and equitable per capita funding are needed in remote minority areas for education, particularly for girls, and for infrastructure and basic services such as sanitation. While China is on target for achieving Millennium Development Goals 4 and 5, focus must now shift to the population pockets with high mortality that are the hardest to study and the hardest to reach with care. This survey reveals that the remote Qinghai Tibetans of Surmang are such a population, whose health status is in danger of remaining obscured by steadily improving Chinese health indicators. Though rebuilding the hospitals in Yushu is a priority, these services will continue to be under utilized unless a strong CHW program is established to provide basic antenatal, delivery, neonatal and postpartum services and to link this under-educated and isolated rural Tibetan population to a network of clinics able to provide basic maternity and child health services.

## List of abbreviations

GDP: gross domestic product; EmOC: emergency obstetric care; MMR: maternal mortality ratio; NGO: non-governmental organization; CHW: community health worker; UN: United Nations.

## Competing interests

The authors declare that they have no competing interests.

## Authors' contributions

MWe analyzed the data and drafted the manuscript. ACL participated in the study design, survey implementation, data collection, data analysis and assisted in drafting the manuscript. KD and MF mapped the survey population and designed the survey. MWi assisted in drafting the manuscript.

SD, PD, KL, DK, JY and OJ participated in study design and conducted the survey.

LM supervised human subjects review board and assisted in drafting the manuscript.

LW is founder of Surmang Foundation and participated in the data interpretation.

All authors read and approved the final manuscript

## Authors Information

Many volunteers from around the globe participated in this effort. LW founded the Surmang Foundation, which built the clinic in Surmang, Yushu in 1992 and is currently working with the Yushu Prefecture Public Health Department to rebuild area clinics in the aftermath of the earthquake. DK is a local primary school teacher. Per recent email, she reports, "We are still in tents but safe. Rebuilding work is going, hope we have brighter day tomorrow." MF's work funded by AJWS.
